# Supplementary material for: Plasmodium falciparum egress disrupts endothelial junctions and activates JAK-STAT signaling in a microvascular 3D blood-brain barrier model
Source: Nat Commun. 2025 Aug 6;16:7262. doi: 10.1038/s41467-025-62514-2 (PMC12328663; doi:10.1038/s41467-025-62514-2)
Supplement: Supplementary file 1 — Supplementary Information [file 41467_2025_62514_MOESM1_ESM.pdf]

Supplementary Information for

Plasmodium falciparum egress disrupts endothelial junctions and activates JAK-STAT signaling in a microvascular 3D blood-brain barrier model

Livia Piatti[#1], Alina Batzilla[#1,2], Fumio Nakaki[1], Hannah Fleckenstein[1,3], François Korbmacher[1], Rory K.M. Long[1,2], Daniel Schraivogel[4], John A. Hawkins[4], Tais Romero-Uruñuela[1†], Borja López-Gutiérrez[1], Silvia Sanz[1], Yannick Schwab[3], Lars M. Steinmetz[4,5,6], James Sharpe[1,7], Maria Bernabeu[1*]

[1] European Molecular Biology Laboratory (EMBL) Barcelona, Barcelona, Spain
[2] Collaboration for joint PhD degree between EMBL and Heidelberg University, Faculty of Biosciences, Heidelberg, Germany
[3] European Molecular Biology Laboratory (EMBL), Cell Biology and Biophysics Unit, Heidelberg, Germany
[4] European Molecular Biology Laboratory (EMBL), Genome Biology Unit, Heidelberg, Germany
[5] Department of Genetics, Stanford University School of Medicine, Stanford, CA, USA.
[6] Stanford Genome Technology Center, Palo Alto, CA, USA.
[7] Institució Catalana de Recerca i Estudis Avançats (ICREA), Barcelona, Spain.

[#] These authors contributed equally to this work

[†] current affiliation tais.romero@isglobal.org

* Correspondence: maria.bernabeu@embl.es

Supplementary Figures 1-5

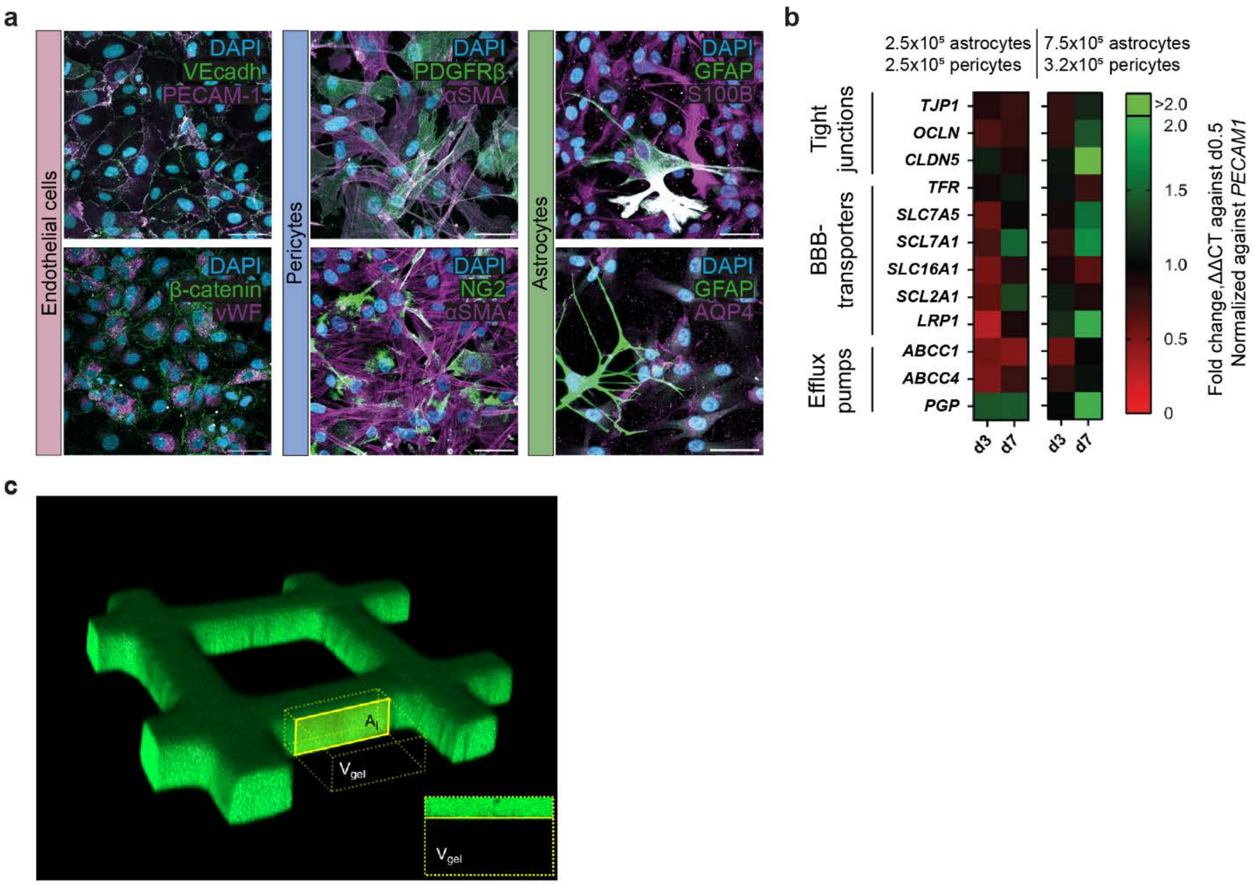

$$P = \frac{1}{\Delta t}\frac{V_{gel}}{A_v}\frac{(I_{gel_1} - I_{gel_0})}{(I_{v_0} - I_{gel_0})}$$

Supplementary Figure 1 – BBB cell types express cell-specific markers and their co-culture induces increased expression of BBB markers over time. a, Representative maximum z-projection of confocal images showing endothelial monolayers expressing VE-cadherin and β-catenin (green), PECAM-1 and vWF (magenta); pericyte monolayers expressing PDGFRβ and NG2 (green), and αSMA (magenta); astrocyte monolayers expressing GFAP (green), S100B and AQP4 (magenta). Nuclei were labeled with DAPI. Scale bar = 50 µm. **b**, Transcriptional expression levels of BBB-specific transporters and junctional markers over time measured by qPCR in 3D-BBB microvessels including a 1:1 or 7:3 astrocyte-to-pericyte ratio. **c**, Representative 3D reconstruction depicting the volume used to measure permeability in a representative 3D-BBB microvessel, shown in the inlet as a maximum z- projection (top). Equation used for permeability quantification after 70 kDa FITC-dextran perfusion (bottom). Source data are provided as a Source Data file.

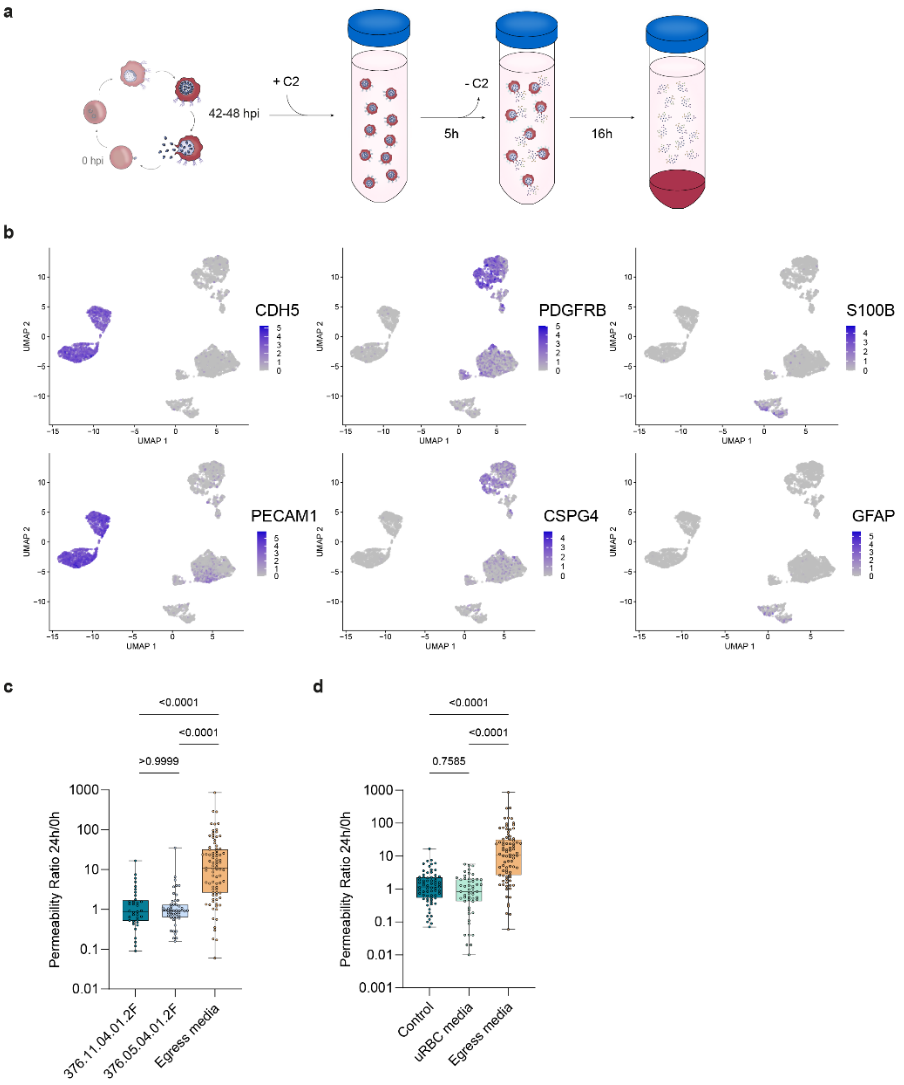

Supplementary Figure 2 – Functional and transcriptional effects of iRBC-egress media on the 3D-BBB model. a, Schematic representation of iRBC-egress media preparation protocol. **b,** UMAP of cells colored by expression of cell type markers for endothelial cells (*CDH5*, *PECAM1*), pericytes (*PDGFRB*, *CSPG4*), and astrocytes (*S100B*, *GFAP*). **c**, Ratio between apparent permeability quantified at 24-hour post-incubation with control media or iRBC-egress media and baseline permeability before incubation, comparing two different HBMEC donors. Each point represents a different ROI from 3D-BBB microvessel models including HBMEC from Lot #376.11.04.01.2F (N = 3) or Lot #376.05.04.01.2F (N = 3), or microvessels incubated with iRBC-egress media (N = 10) (Kruskal Wallis with Dunn's multiple comparisons test). **d**, Ratio between apparent permeability quantified at 24-hour post-incubation with control media, uRBC media or iRBC-egress media and baseline permeability before incubation. Each point represents a different ROI from 3D-BBB microvessel models exposed to control media (N = 3), uRBC media (N = 4), or iRBC-egress media (N = 10) (Kruskal Wallis with Dunn's multiple comparisons test). Box plots display the median (line within the box), interquartile range (box), and range (whiskers). Source data are provided as a Source Data file.

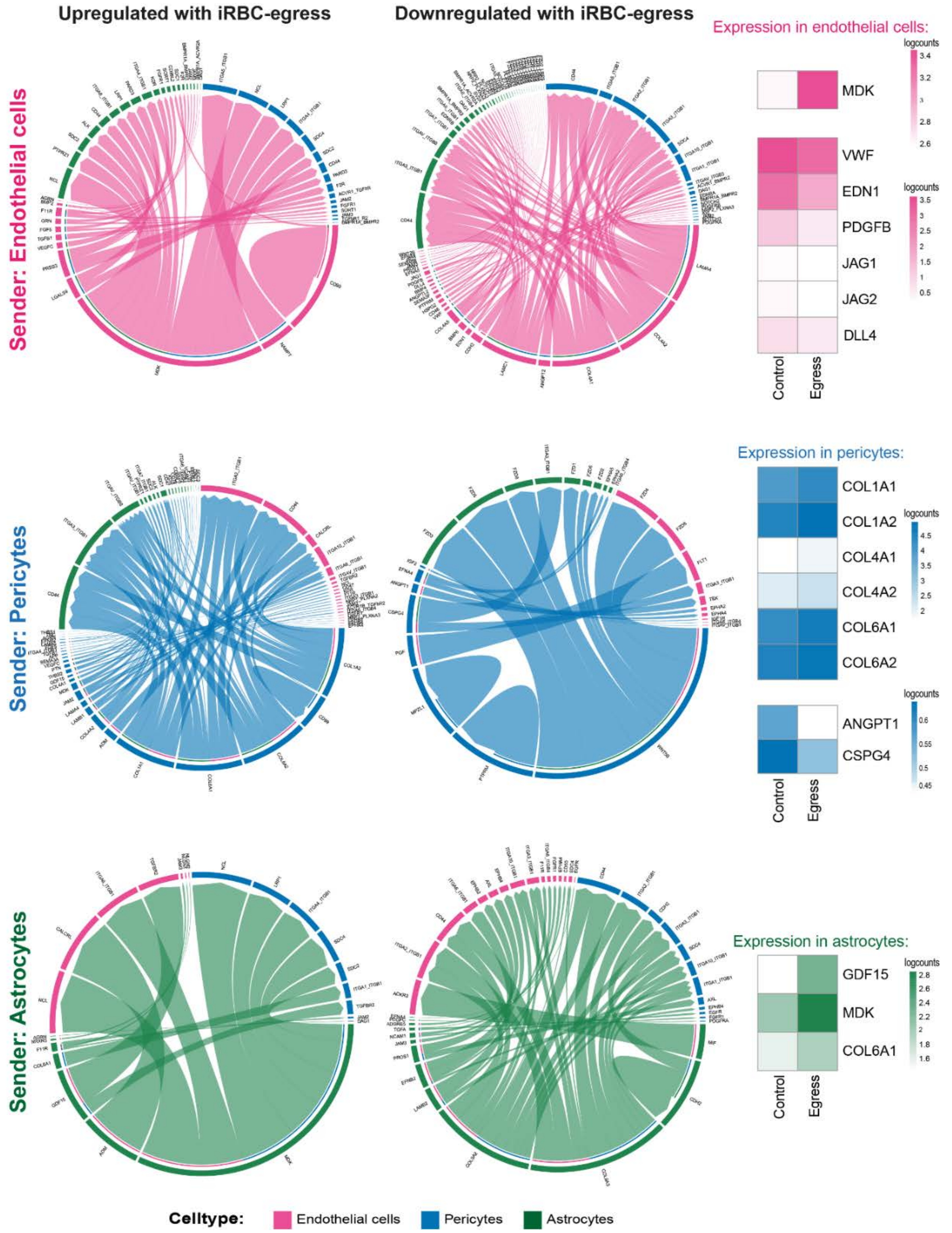

Supplementary Figure 3 – Altered BBB cell ligand-receptor interactions upon incubation with iRBC-egress media. Significantly up- and down-regulated ligand-receptor interactions identified after exposure to iRBC-egress between the three BBB cell types using the *CellChat* package. Arrows point from ligands on sender cells to receptors on receiver cells and are colored by sender cell. Weights of links are proportional to the interaction strength. Heatmaps show log2-transformed normalized expression values of genes of selected BBB-specific interactions in the respective cell types.

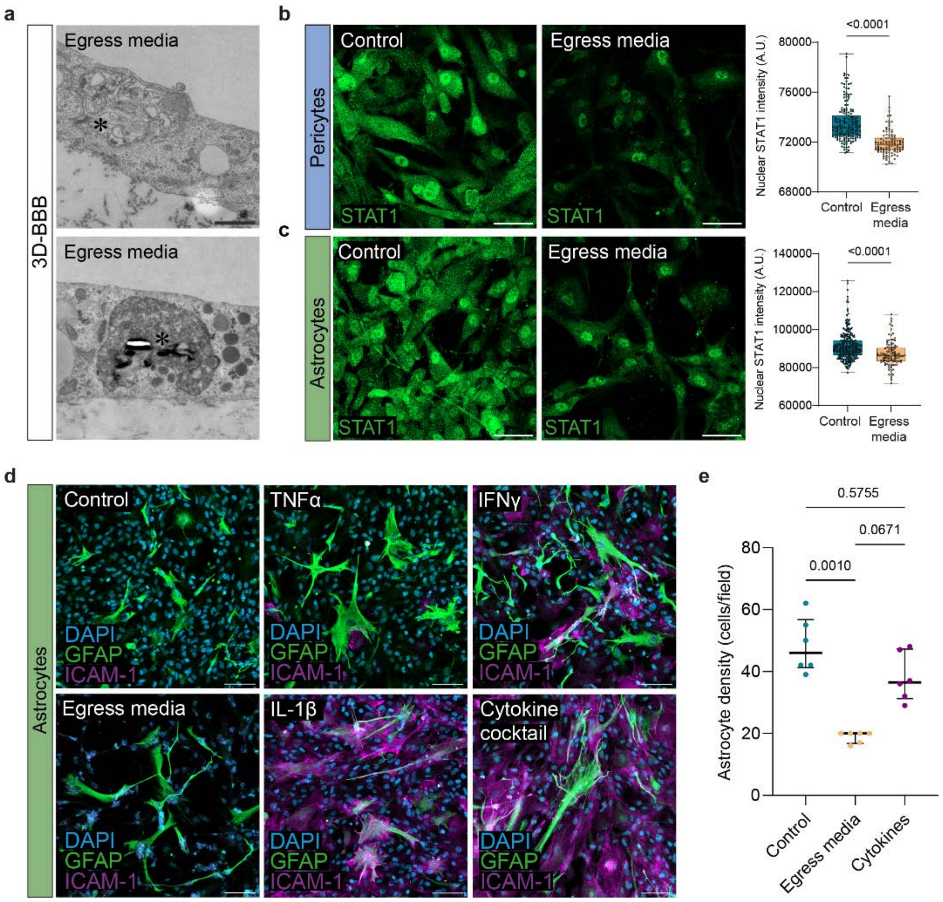

Supplementary Figure 4 – Activation of inflammatory response in 3D-BBB microvessels and 2D monolayers upon incubation with iRBC-egress media or cytokines. a, TEM images of endothelial cells within the 3D-BBB microvessels after incubation with iRBC-egress media, showing vacuoles containing iRBC membranes (top) and electron-dense material with hemozoin crystals (bottom). Scale bar = 1 µm. **b,** Representative maximum z-projection of confocal images showing STAT1 protein localization (green) and relative nuclear mean fluorescence intensity in pericytes monolayers (N = 3/condition) after 24-hour incubation with iRBC-egress media or media control (Two-tailed Mann-Whitney U test). Box plots display the median (line within the box), interquartile range (box), and range (whiskers). Scale bar = 50 µm. **c**, Representative maximum z-projection of confocal images showing STAT1 protein localization (green) and relative nuclear mean fluorescence intensity in astrocytes monolayers (N = 3/condition) after 24-hour incubation with iRBC-egress media or media control (Two-tailed Mann-Whitney U test). Box plots display the median (line within the box), interquartile range (box), and range (whiskers). Scale bar = 50 µm. **d,** Representative maximum z-projection of confocal images showing the expression of GFAP (green) and ICAM-1 (magenta) in 2D astrocyte monolayers incubated with media control, iRBC-egress media, TNFα, IFNγ and IL-1β, or a cytokine cocktail for 24 hours. Scale bar = 100 µm. **e**, Quantification of astrocyte cell density comparing 2D monolayers incubated with control media, iRBC-egress media or a cytokine cocktail for 24 hours (Kruskal-Wallis with Dunn's multiple comparisons test). The median value is reported as a line, with error bars indicating the interquartile range. Source data are provided as a Source Data file.

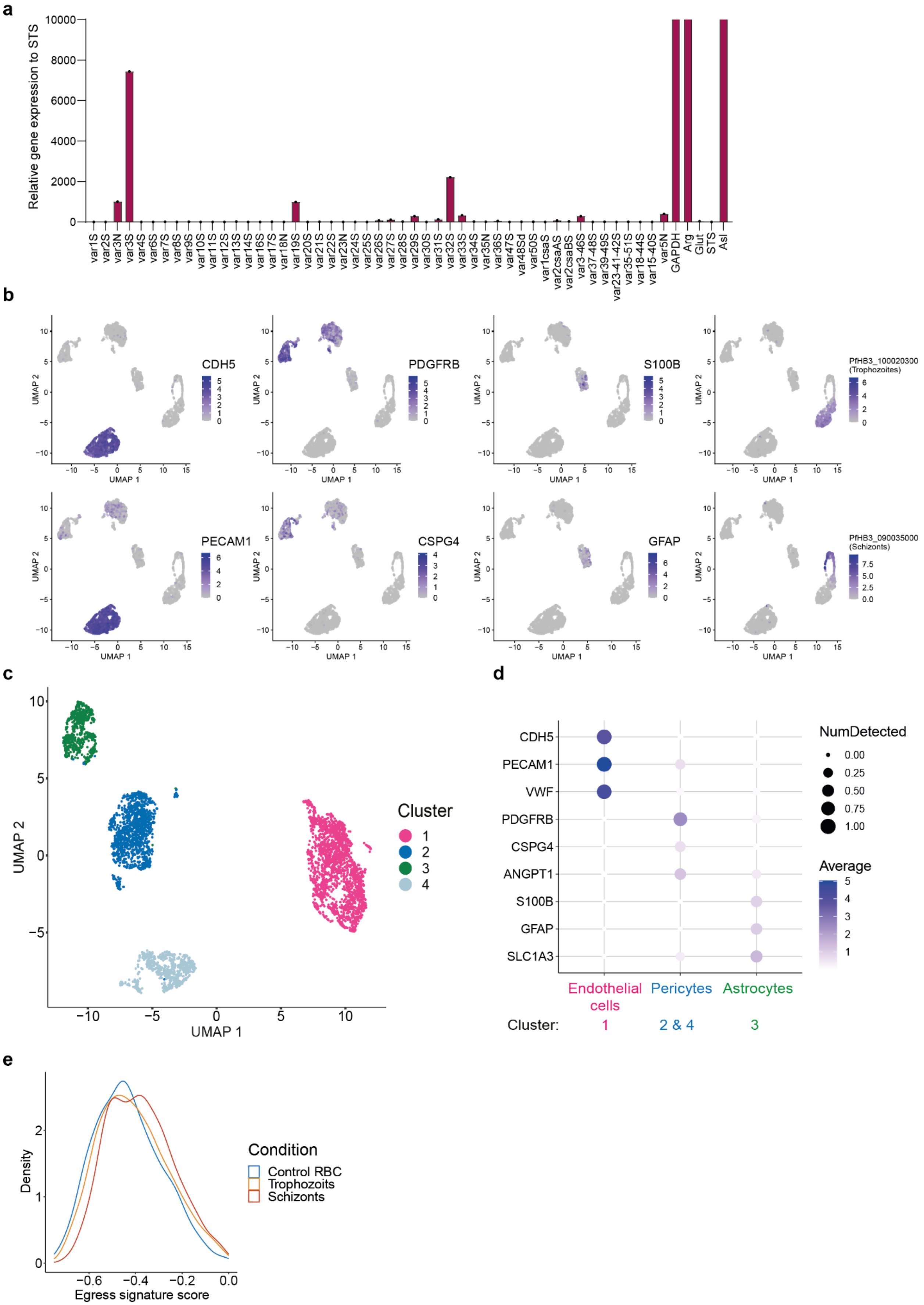
a
Relative gene expression to STS
var1S var2S var3N var3S var4S var6S var7S var8S var9S var10S var11S var12S var13S var14S var16S var17S var18N var19S var20S var21S var22S var23N var24S var25S var26S var27S var28S var29S var30S var31S var32S var33S var34S var35N var36S var47S var48Sd var50S var1csaS var2csaAS var2csaBS var3-46S var37-48S var39-49S var23-41-42S var35-51S var18-44S var15-40S var5N GAPDH Arg Glut STS Asl
b
CDH5
PDGFRB
S100B
PfHB3_100020300 (Trophozoites)
PECAM1
CSPG4
GFAP
PfHB3_090035000 (Schizonts)
UMAP 1
UMAP 2
c
Cluster
UMAP 1
UMAP 2
d
CDH5
PECAM1
VWF
PDGFRB
CSPG4
ANGPT1
S100B
GFAP
SLC1A3
NumDetected
Average
Endothelial cells
Pericytes
Astrocytes
Cluster:
1
2 & 4
3
e
Density
Egress signature score
Condition
Control RBC
Trophozoits
Schizonts

Supplementary Figure 5 – scRNA-seq analysis after perfusion of 3D-BBB microvessels with *P. falciparum*-iRBC trophozoites and schizonts. a, Relative gene expression levels of PfEMP1 variants in the *P. falciparum* line HB3var03. Source data are provided as a Source Data file. **b,** UMAP of cells colored by expression of cell type marker for endothelial cells (*CDH5*, *PECAM1*), pericytes (*PDGFRB, CSPG4*), astrocytes (*S100B, GFAP*), trophozoites (*PfHB3_100020300, PFHG-02607*), and schizonts (*PfHB3_090035000, PFHG-03202*). **c**, UMAP of filtered BBB cells colored by unsupervised *Leiden* clustering. **d**, Dot plot of the expression of cell type specific markers in the respective clusters. **e**, Density plot showing the distribution of the *egress signature score* in endothelial cells exposed to control RBC, trophozoites, or schizonts.
