# Supplementary material for: Plasmodium falciparum egress disrupts endothelial junctions and activates JAK-STAT signaling in a microvascular 3D blood-brain barrier model
Source: Nat Commun. 2025 Aug 6;16:7262. doi: 10.1038/s41467-025-62514-2 (PMC12328663; doi:10.1038/s41467-025-62514-2)
Supplement: Supplementary file 2 — Description of additional supplementary files [file 41467_2025_62514_MOESM2_ESM.pdf]

Description of additional supplementary files

Supplementary Data 1

Significantly differentially expressed genes in all cell types and conditions identified using the hurdle (two-part generalized regression) model from the MAST package. P-values, logfoldchange values, and false discovery rate (FDR) obtained from Benjamini-Hochberg method are reported.

Supplementary Data 2

Significant GO-terms for HBMEC in iRBC-egress, schizont and trophozoite conditions and for pericytes and astrocytes in iRBC-egress condition. P-values, Benjamini-Hochberg adjusted p-values, and upregulated genes in respective GO-term are provided.

Supplementary Data 3

List of oligonucleotides used as primer sequences in qPCR experiments on 3D-BBB microvessels or the *P. falciparum* HB3var03 line.
