# Supplementary material for: Plasmodium falciparum egress disrupts endothelial junctions and activates JAK-STAT signaling in a microvascular 3D blood-brain barrier model
Source: Nat Commun. 2025 Aug 6;16:7262. doi: 10.1038/s41467-025-62514-2 (PMC12328663; doi:10.1038/s41467-025-62514-2)
Supplement: Supplementary file 6 — Reporting summary [file 41467_2025_62514_MOESM6_ESM.pdf]

nature portfolio

Corresponding author(s): Maria Bernabeu

Last updated by author(s): 08/07/2025

Reporting Summary

Nature Portfolio wishes to improve the reproducibility of the work that we publish. This form provides structure for consistency and transparency in reporting. For further information on Nature Portfolio policies, see our Editorial Policies and the Editorial Policy Checklist.

Statistics

For all statistical analyses, confirm that the following items are present in the figure legend, table legend, main text, or Methods section.

n/a	Confirmed	
☐	☒	The exact sample size (*n*) for each experimental group/condition, given as a discrete number and unit of measurement
☐	☒	A statement on whether measurements were taken from distinct samples or whether the same sample was measured repeatedly
☐	☒	The statistical test(s) used AND whether they are one- or two-sided<br>*Only common tests should be described solely by name; describe more complex techniques in the Methods section.*
☒	☐	A description of all covariates tested
☐	☒	A description of any assumptions or corrections, such as tests of normality and adjustment for multiple comparisons
☐	☒	A full description of the statistical parameters including central tendency (e.g. means) or other basic estimates (e.g. regression coefficient) AND variation (e.g. standard deviation) or associated estimates of uncertainty (e.g. confidence intervals)
☐	☒	For null hypothesis testing, the test statistic (e.g. *F*, *t*, *r*) with confidence intervals, effect sizes, degrees of freedom and *P* value noted<br>*Give P values as exact values whenever suitable.*
☒	☐	For Bayesian analysis, information on the choice of priors and Markov chain Monte Carlo settings
☒	☐	For hierarchical and complex designs, identification of the appropriate level for tests and full reporting of outcomes
☒	☐	Estimates of effect sizes (e.g. Cohen's *d*, Pearson's *r*), indicating how they were calculated

Our web collection on statistics for biologists contains articles on many of the points above.

Software and code

Policy information about availability of computer code

Data collection	- Confocal images were acquired using a LSM980 Airyscan 2 microscope (Zeiss) and processed with imaging software ZEN (Zeiss, v3.3.89) and Fiji (ImageJ, v1.54f).<br>- Electron microscopy: PELCO Biowave Pro microwave processor (Ted Pella, Inc.) containing a SteadyTemp Pro and a ColdSpot. Acquisition on a JEOL JEM 2100 plus at 80 or 120 keV using SerialEM.<br>- Single cell RNA sequencing: Chromium Controller (10x Genomics, firmware version 4.00). Sequencing with NextSeq2000 (Illumina).
Data analysis	- Confocal images were processed with imaging software ZEN (Zeiss, v3.3.89), Fiji (ImageJ, v1.54f) and Vision4D (Arivis, v3.5.1).<br>- Electron microscopy: Montages were processed using IMOD's Blend Montages function and Fiji (ImageJ, v1.54f).<br>- Statistical analysis: GraphPad Prism (version 10.2.0), R (version 4.2.2)<br>- Single cell analysis: R (v4.2.2), CellRanger (v. 7.0.1). We used custom R scripts with R packages: deMULTIplex (v.1.0.2), scuttle (v.1.8.4), scDblFinder (v. 1.10.0), scran (v.1.24.1), scater (v.1.26.1), bluster (v.1.8.0),batchelor (v.1.14.1), MAST (v.1.22.0), pheatmap (v.1.0.12), dendextend (v.1.16.0), clusterprofiler (v.4.4.4), GOSemSim (v.2.22.0), enrichplot (v.1.16.2), tidySingleCellExperiment (v.1.6.3), PROGENy (v.1.18.0), CellChat (v.1.6.1)<br>Custom codes were deposited to GitHub [github.com/Alina-Ba/scRNAseq_iRBC]

For manuscripts utilizing custom algorithms or software that are central to the research but not yet described in published literature, software must be made available to editors and reviewers. We strongly encourage code deposition in a community repository (e.g. GitHub). See the Nature Portfolio guidelines for submitting code & software for further information.

Data

Policy information about availability of data

All manuscripts must include a data availability statement. This statement should provide the following information, where applicable:

- Accession codes, unique identifiers, or web links for publicly available datasets
- A description of any restrictions on data availability
- For clinical datasets or third party data, please ensure that the statement adheres to our policy

The scRNA-seq data used in this study are available in the ArrayExpress database under accession code E-MTAB-14463. Source data are provided with this paper.

Research involving human participants, their data, or biological material

Policy information about studies with human participants or human data. See also policy information about sex, gender (identity/presentation), and sexual orientation and race, ethnicity and racism.

Reporting on sex and gender	*Use the terms sex (biological attribute) and gender (shaped by social and cultural circumstances) carefully in order to avoid confusing both terms. Indicate if findings apply to only one sex or gender; describe whether sex and gender were considered in study design; whether sex and/or gender was determined based on self-reporting or assigned and methods used. Provide in the source data disaggregated sex and gender data, where this information has been collected, and if consent has been obtained for sharing of individual-level data; provide overall numbers in this Reporting Summary. Please state if this information has not been collected. Report sex- and gender-based analyses where performed, justify reasons for lack of sex- and gender-based analysis.*
Reporting on race, ethnicity, or other socially relevant groupings	*Please specify the socially constructed or socially relevant categorization variable(s) used in your manuscript and explain why they were used. Please note that such variables should not be used as proxies for other socially constructed/relevant variables (for example, race or ethnicity should not be used as a proxy for socioeconomic status). Provide clear definitions of the relevant terms used, how they were provided (by the participants/respondents, the researchers, or third parties), and the method(s) used to classify people into the different categories (e.g. self-report, census or administrative data, social media data, etc.) Please provide details about how you controlled for confounding variables in your analyses.*
Population characteristics	*Describe the covariate-relevant population characteristics of the human research participants (e.g. age, genotypic information, past and current diagnosis and treatment categories). If you filled out the behavioural & social sciences study design questions and have nothing to add here, write "See above."*
Recruitment	*Describe how participants were recruited. Outline any potential self-selection bias or other biases that may be present and how these are likely to impact results.*
Ethics oversight	*Identify the organization(s) that approved the study protocol.*

Note that full information on the approval of the study protocol must also be provided in the manuscript.

Field-specific reporting

Please select the one below that is the best fit for your research. If you are not sure, read the appropriate sections before making your selection.

☒ Life sciences ☐ Behavioural & social sciences ☐ Ecological, evolutionary & environmental sciences

For a reference copy of the document with all sections, see nature.com/documents/nr-reporting-summary-flat.pdf

Life sciences study design

All studies must disclose on these points even when the disclosure is negative.

Sample size	We have reported the number of samples used in each experiment in the figure legends.
Data exclusions	3D-BBB microvessels were excluded from the experiments if flow was unstable during live imaging or parasite perfusion.
Replication	All replication attempts were successful, with variability detailed in the graphs and figure legends.
Randomization	*Describe how samples/organisms/participants were allocated into experimental groups. If allocation was not random, describe how covariates were controlled OR if this is not relevant to your study, explain why.*
Blinding	*Describe whether the investigators were blinded to group allocation during data collection and/or analysis. If blinding was not possible, describe why OR explain why blinding was not relevant to your study.*

Reporting for specific materials, systems and methods

We require information from authors about some types of materials, experimental systems and methods used in many studies. Here, indicate whether each material, system or method listed is relevant to your study. If you are not sure if a list item applies to your research, read the appropriate section before selecting a response.

Materials & experimental systems

n/a	Involved in the study
☐	☒ Antibodies
☐	☒ Eukaryotic cell lines
☒	☐ Palaeontology and archaeology
☒	☐ Animals and other organisms
☒	☐ Clinical data
☒	☐ Dual use research of concern
☒	☐ Plants

Methods

n/a	Involved in the study
☒	☐ ChIP-seq
☒	☐ Flow cytometry
☒	☐ MRI-based neuroimaging

Antibodies

Antibodies used

Primary Ab:
-vWF (Bio-Rad AHP062, 1:200)
-PECAM1 (BD Pharmingen 560983, 1:100),
-β-catenin (Santa Cruz Biotechnology sc-59737, 1:200),
-ICAM-1 (Abcam ab20, 1:200),
-GFAP (Abcam ab4674, 1:200),
-S100B (Sigma S2532-100U, 1:200),
-AQP4 (Novus Biologicals NBP1-87679, 1:200),
-αSMA (Abcam ab202509, 1:200),
-PDGFRβ (Abcam ab69506, 1:200),
-NG2 (Invitrogen 372700, 1:200),
-GFP (Invitrogen A21311, 1:200),
-mCherry (Invitrogen M11240, 1:200),
-VE-cadherin (Santa Cruz Biotechnology sc-52751, 1:100 or Abcam ab33168, 1:100),
-STAT1 (Cell Signaling 14994S, 1:100),
-LAMP1 (Cell Signaling 9091, 1:100),
-ZO-1 (Invitrogen 339100, 1:100).

Secondary Ab:
-Alexa-Fluor 488-conjugated secondary antibodies (Invitrogen, 1:250)
-Alexa-Fluor 594-conjugated secondary antibodies (Invitrogen, 1:250)
-Alexa-Fluor 647-conjugated secondary antibodies (Invitrogen, 1:250)

Validation

For all antibodies used in this study, we relied on the validation provided by the manufacturers, as reported on their respective websites, and selected antibodies with established use in the literature for the relevant applications.
- vWF (Bio-Rad AHP062) https://www.bio-rad-antibodies.com/polyclonal/human-von-willebrand-factor-antibody-ahp062.html?f=purified
-PECAM1 (BD Pharmingen 560983) https://www.bdbiosciences.com/en-de/products/reagents/flow-cytometry-reagents/research-reagents/single-color-antibodies-ruo/pe-mouse-anti-human-cd31.560983?tab=product_details
-β-catenin (Santa Cruz Biotechnology sc-59737) https://www.scbt.com/p/beta-catenin-antibody-12f7?srsltid=AfmBOopR1ysQlndpuWAJe1LUkR7zgT-wm2mte4PyA3dVp-jGZBkVEwAc
-ICAM-1 (Abcam ab20) https://www.abcam.com/en-us/products/primary-antibodies/icam1-antibody-152-ab20?srsltid=AfmBOopirtdr8yU3TW-IFjxUb99nHi1jH94hOqdR4KFEx2R0gqPrJqTc
-GFAP (Abcam ab4674) https://www.abcam.com/en-us/products/primary-antibodies/gfap-antibody-astrocyte-marker-ab4674?srsltid=AfmBOopCOcC476GpmnYXi79KPOUyRP1wc2a8TUeSmTtqtPxMGmaSRv2u
-S100B (Sigma S2532-100U) https://www.sigmaaldrich.com/ES/es/product/sigma/s2532?srsltid=AfmBOorsQY29MHShiBHX9jAiN8SKB2yhT_rgiRjogwAfRF3xDPXnKKny
-AQP4 (Novus Biologicals NBP1-87679) https://www.novusbio.com/products/aquaporin-4-antibody_nbp1-87679?srsltid=AfmBOoqUIEZ74T6J1DW9BtA21owzaYwob4f95kM1IEeYOCCMzQAPv5nN
-αSMA (Abcam ab202509) https://www.abcam.com/en-us/products/primary-antibodies/alexa-fluor-555-alpha-smooth-muscle-actin-antibody-epr5368-ab202509?srsltid=AfmBOoqdQDiAyS6LDced0z9DNKTD-hjng8iSPM_7hLLwWRInL92quwch
-PDGFRβ (Abcam ab69506) https://www.abcam.com/en-us/products/primary-antibodies/pdgfr-beta-antibody-42g12-ab69506?srsltid=AfmBOor54_0ExTnbkf-ThnBNpYZe8bMH6MFxLB4_4pw07CGs_s3l-_SG
-NG2 (Invitrogen 372700) https://www.thermofisher.com/antibody/product/NG2-Antibody-clone-D120-43-D4-11-N143-8-N109-6-Monoclonal/37-2700
-GFP (Invitrogen A21311) https://www.thermofisher.com/antibody/product/GFP-Antibody-Polyclonal/A-21311
-mCherry (Invitrogen M11240) https://www.thermofisher.com/antibody/product/mCherry-Antibody-clone-16D7-Monoclonal/M11240
-VE-cadherin (Santa Cruz Biotechnology sc-52751) https://www.scbt.com/p/ve-cadherin-antibody-bv9?srsltid=AfmBOoouHoBkLUYXvIP9LOxvfPhqoHSqBlNUwEKN2Ul16HyTRjlMnrQx

-VE-cadherin (Abcam ab33168) https://www.abcam.com/en-us/products/primary-antibodies/ve-cadherin-antibody-intercellular-junction-marker-ab33168?srsltid=AfmBOoqvR8rPYWoyE6aAghbF6O2xLmHW_6orYRR8DiJCU6V6RzHkPkCC
-STAT1 (Cell Signaling Technology 14994S) https://www.cellsignal.com/products/primary-antibodies/stat1-d1k9y-rabbit-mab/14994?srsltid=AfmBOoqreHv8LJX7bQ9dnh3YD1OZ9VUxr8Jh6LMv3qpMb5n5hnhnFQUz
-LAMP1 (Cell Signaling Technology 9091) https://www.cellsignal.com/products/primary-antibodies/lamp1-d2d11-xp-rabbit-mab/9091?srsltid=AfmBOopXXMmNLyLAsGGzbK2TYEDZmiivxMceujeF1Vb7hhRpntH6LsI-
-ZO-1 (Invitrogen 339100) https://www.thermofisher.com/antibody/product/ZO-1-Antibody-clone-ZO1-1A12-Monoclonal/33-9100

Secondary Ab:
-Alexa-Fluor 488-conjugated secondary antibodies (Invitrogen, 1:250) https://www.thermofisher.com/antibody/secondary/query/alexa%20fluor%20488
-Alexa-Fluor 594-conjugated secondary antibodies (Invitrogen, 1:250) https://www.thermofisher.com/antibody/secondary/query/alexa%20fluor%20594
-Alexa-Fluor 647-conjugated secondary antibodies (Invitrogen, 1:250) https://www.thermofisher.com/antibody/secondary/query/alexa%20fluor%20647

Eukaryotic cell lines

Policy information about cell lines and Sex and Gender in Research

Cell line source(s)
- HBMEC, Lot 376.05.04.01.2F or 376.11.04.01.2F, Cell Systems.
- HA, Lot 31978, ScienCell.
- HBVP, Lot 32562, ScienCell

Authentication
Immunofluorescence of main cell type markers.

Mycoplasma contamination
Mycoplasma PCR was performed every 2 weeks.

Commonly misidentified lines (See ICLAC register)
Name any commonly misidentified cell lines used in the study and provide a rationale for their use.

Plants

Seed stocks
Report on the source of all seed stocks or other plant material used. If applicable, state the seed stock centre and catalogue number. If plant specimens were collected from the field, describe the collection location, date and sampling procedures.

Novel plant genotypes
Describe the methods by which all novel plant genotypes were produced. This includes those generated by transgenic approaches, gene editing, chemical/radiation-based mutagenesis and hybridization. For transgenic lines, describe the transformation method, the number of independent lines analyzed and the generation upon which experiments were performed. For gene-edited lines, describe the editor used, the endogenous sequence targeted for editing, the targeting guide RNA sequence (if applicable) and how the editor was applied.

Authentication
Describe any authentication procedures for each seed stock used or novel genotype generated. Describe any experiments used to assess the effect of a mutation and, where applicable, how potential secondary effects (e.g. second site T-DNA insertions, mosiacism, off-target gene editing) were examined.
